# Compound heterozygous variants in *MYH11* underlie autosomal recessive megacystis-microcolon-intestinal hypoperistalsis syndrome in a Chinese family

**DOI:** 10.1038/s10038-019-0651-z

**Published:** 2019-08-19

**Authors:** Qin Wang, Jianming Zhang, Hui Wang, Qing Feng, Fuwei Luo, Jiansheng Xie

**Affiliations:** Affiliated Shenzhen Maternity and Child Healthcare Hospital, Southern Medical University, No. 3012, Fuqiang Road, 518028 Guangdong, Shenzhen China

**Keywords:** Medical genetics, Genetics research

## Abstract

Megacystis-microcolon-intestinal-hypoperistalsis syndrome (MMIHS) is a rare and severe disorder characterized by functional obstruction in the urinary and gastrointestinal tract. The molecular basis of this condition has been defined recently. Heterozygous variants in *ACTG2*, homozygous mutations in *LMOD1, MYLK*, and *MYH9* were related to the pathogenesis of the syndrome, which encodes proteins involved in the process of smooth muscle contraction, supporting a myopathic basis for the disease. Recent studies have identified homozygous or compound heterozygous variants in *MYH11* as a candidate gene of MMIHS. In this report, we described a nonconsanguineous Chinese family with three male fetuses affected with megacystis. Trio-targeted exome sequencing identified compound heterozygous variants, c.2051 G > A (p.R684H) and c.3540_3541delinsTT (p.(E1180D, Q1181Ter)), in *MYH11* (NM_001040114). The variants were inherited from the parents, respectively. Western blotting showed a marked decrease in *MYH11* protein in the proband’s umbilical cord tissue compared with the control sample. The study’s results confirmed that *MYH11* is a candidate gene for MMIHS with autosomal recessive (AR) inheritance and expanded the mutation spectrum for this clinical condition. Combining clinical phenotype with molecular diagnosis may enable the identification of candidate genes for potential monogenic diseases and facilitate accurate genetic counseling, informed decision-making, and prenatal diagnosis.

## Introduction

Megacystis-microcolon-intestinal-hypoperistalsis syndrome (MMIHS) is a rare congenital condition that is characterized by prenatal bladder enlargement, neonatal functional gastrointestinal obstruction, and chronic dependence on total parenteral nutrition (TPN) and urinary catheterization [1, 2]. This condition was first described in 1976 by Berdon, and subsequently, a total of 450 patients with MMIHS have been reported [3–5]. MMIHS is a severe form of functional intestinal obstruction in the newborn, and the majority of the cases succumb to a fatal outcome due to sepsis followed by multiple organ failure and malnutrition [2, 6]. Various hypotheses have been proposed regarding the pathogenesis of MMIHS, including genetic, neurogenic, myogenic, and hormonal origins [2].

The etiology of MMIHS is heterozygous, and most cases are autosomal-dominant sporadic and caused by de novo heterozygous variants in the *ACTG2* (actin, gamma 2) gene, which encodes actin gamma as a component of the cytoskeleton and a mediator of internal cell motility [7]. Recent reports in the offspring of consanguineous families have proposed the AR inheritance in MMIHS*. LMOD1* (leiomodin 1), a gene preferentially expressed in vascular and visceral smooth muscle cells, is involved in MMIHS caused by a homozygous premature termination mutation [8]. *MYLK* (myosin light chain kinase), encoding an important kinase required for myosin activation and subsequent interaction with actin filaments, is related to the recessive form of MMIHS [9]. A homozygous deletion in *MYL9* (myosin light chain 9), which encodes a myosin light chain, is a candidate gene for the AR form of MMIHS [10]. A homozygous mutation in a consanguineous family, compound heterozygous mutations and a heterozygous variant with a 16p13.11 microdeletion in nonconsanguineous family in *MYH11* (myosin heavy chain 11) have been reported in MMIHS [11–13]. These five genes related to MMIHS are involved in the smooth muscle contraction, and the functional study of proteins supports a myopathic basis for this clinical condition.

At present, there is no specific treatment for MMIHS, and management for affected newborns remains a challenge for doctors and parents. The survivors were either maintained by TPN or had undergone multivisceral transplantation. With the increasing knowledge on the pathogenesis of MMIHS, prenatal diagnosis for this syndrome is necessary and crucial for genetic counseling. The most common prenatal finding of MMIHS is a large, progressive distended bladder associated with polyhydramnios or normal amniotic fluid volume detected by ultrasonography. Hydronephrosis is noted, and the intestine usually appears normal or is dilated in some cases [1, 14]. Fetal urine biochemical markers can be helpful for the differentiation of MMIHS from posterior urethral valves or other megacystis [15, 16]. Exome sequencing is rapidly evolving and has demonstrated potential clinical utility in the identification of new disease-causing genes for Mendelian disease [17, 18]. In this study, we present the detection of compound heterozygous variants, c.2051 G > A (p.R684H) and c.3540_3541delinsTT(p.(E1180D,Q1181Ter)), in *MYH11* (NM_001040114) in three consecutive male fetuses with MMIHS in a Chinese family. The variants were inherited from the parents and were confirmed by Sanger sequencing. *MYH11* c.2051 G > A (p.R684H) has been registered in the dbSNP as rs1478913138 (T = 0.00000, 1/245930, Genome Aggregation Database) and c.3540_3541delinsTT (p.(E1180D, Q1181Ter)) is a novel heterozygous variant. Western blotting showed a marked decrease in MYH11 protein in the proband’s umbilical cord tissue compared with the control sample, which demonstrated that the variants affect the MYH11 protein expression and that its normal function may be damaged. This result expands the genetic spectrum and supports *MYH11* as a candidate gene for MMIHS with AR pattern of inheritance. More case reports may help to elucidate the function of *MYH11* that may be critical to understanding the genetic etiology of this rare and severe heterogeneity disease.

## Materials and methods

### Subjects

The index fetus is the second pregnancy of a nonconsanguineous couple. The pregnant woman was 29-years-old, G_3_P_0_ (gestation 3, production 0), with no significant past medical, surgical, or family disease history. Physical examinations on the couple were normal. The couple were referred for fetal megacystis at the genetic counseling clinic in Shenzhen Maternity and Child Healthcare Hospital. The couple had three consecutive male fetuses with similar ultrasonic structural anomalies. Their first fetus was observed with an enlarged bladder by ultrasound sonography examination and was terminated at 14 weeks of gestation. Their second fetus was observed with the same ultrasonic structural anomalies and was terminated at 17 weeks of gestation. Fetal umbilical cord tissue was sampled from the second fetus. Peripheral blood was obtained from the parents. The chorionic villus was sampled from the third fetus at 14 weeks of pregnancy. DNA was extracted as previously described [19]. The first fetus was not available for molecular testing. The present study was approved by the hospital’s Institutional Review Board, and written informed consent was obtained from the parents.

### Targeted exome sequencing

The present study used the NextSeq 500/550 Mid Output v2 kit (300 cycles) (Illumina) with a high depth of coverage for 4000 medical exome genes that are associated with clinically relevant phenotypes. The genomic DNA paired-end libraries were constructed based on the manufacturer’s instructions using the NimbleGen SeqCap EZ Choice Library (Roche). The captured DNA libraries were enriched and then sequenced using the Illumina NextSeq550 platform with an overall > 10× coverage depth. The clean reads from the Illumina NextSeq550 were aligned to the human genome reference (hg19/GRCh37). BAM and VCF files were generated by NextGENe software (SoftGenetics, State College, PA). Variants were annotated and filtered by Ingenuity Variant Analysis (https://variants.ingenuity.com). In silico variant prediction analysis were performed via PolyPhen2, Sorting Intolerant From Tolerant, Mutation Taster and Provean.

### Sanger sequencing

Sanger sequencing of compound heterozygous variants in *MYH11* was performed essentially as to confirm the exome panel sequencing results in fetus-mother-father trio samples. The proband’s mother had chorionic villus sampling (CVS) at 14 weeks for the third fetus for Sanger sequencing of compound heterozygous variants. The primers used to amplify the mutant sequence were MYH11-2051-F (5’ ACTTGGAAACCCTGACTGCC 3’) and MYH11-2051-R (5’GCATCCTCCCTTCCTCCTTT3’), MYH11-3540-F (5’TTTGGGTTGCTCTGAGGATT3’), and MYH11-3540-R (5’ AGGCTGCTGATGTCACTCTT3’).

### Protein isolation and Western blotting

Umbilical cord tissue from the index patient and the control sample were washed with PBS and incubated with standard lysis buffer (20 mM Tris (pH7.5), 150 mM NaCl, 1% Triton X-100, P0013, Beyotime Biotechnology, China) for 30 min on ice. Cell lysates were collected by scraping and cleared by centrifugation at 14000 rpm for 10 min at 4 °C. Protein was quantitated using bicinchoninic acid (Thermo Scientific), and 20 µg of each sample was loaded in a 5–10% precast polyacrylamide gel (Bio-Rad). Protein was transferred to nitrocellulose membranes and incubated with antibodies. Anti-MYH11 antibody (1:50, Monoclonal rabbit IgG, BM5659, Boster Biological Technology, China) was used as a primary antibody. Anti-glyceraldehyde 3-phosphate dehydrogenase (GAPDH) loading control (1:10000; ab8245; Abcam) was used as an internal control. The membrane was then incubated with secondary antibody and proteins were visualized using chemiluminescence on X-ray film (Immobilon Western Chemilum HRP substrate, KLS0500, Millipore).

## Results

### Clinical findings

**Fig. 1:**
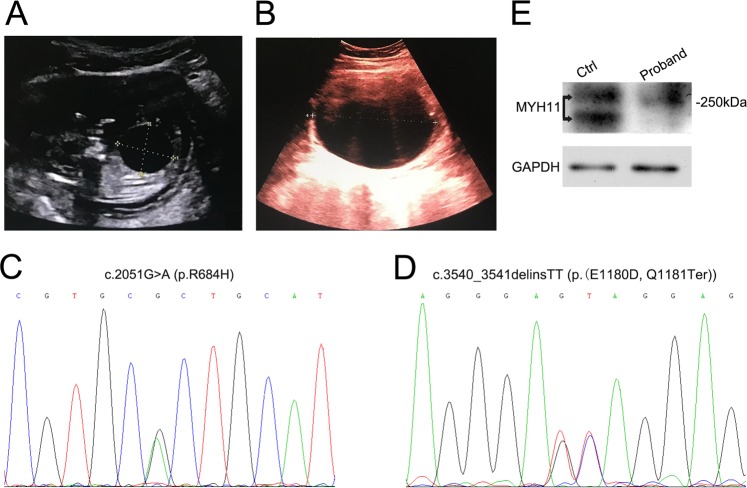
Compound heterozygous variants in *MYH11* in a family with MMIHS. **a** Prenatal ultrasonography image at 13 weeks of gestation for the index fetus demonstrated a distended bladder (2.56 cm × 2.32 cm). **b** Prenatal ultrasonography image at 17 weeks shows a progressive distention of the bladder (9.5 cm × 7.16 cm) in the index fetus. **c** Sanger sequencing validates the exome sequencing variant of c.2051 G > A (p.R684H) in *MYH11* (NM_001040114). **d** Sanger sequencing validates the exome sequencing variant of c.3540_3541delinsTT (p.(E1180D, Q1181Ter)) in *MYH11* (NM_001040114). **e** Protein expression of MYH11 in the control (Ctrl) and proband umbilical cord tissues. Arrows point to the band location for protein MYH11

The couple was nonconsanguineous and physically healthy with no echocardiographic anomalies. Their first fetus was detected with an enlarged bladder by ultrasound screening at 13 weeks of gestation, and the pregnancy was terminated at 14 weeks. Prenatal ultrasonography performed at 13 weeks of gestation for the index fetus identified the presence of a distended bladder (2.56 × 2.32 cm) (Fig. 1a). A progressive distention of the bladder was observed at 17 weeks of gestation (9.5 × 7.16 cm) (Fig. 1b). Oligohydramnios was noted (AFI = 5.5 cm) and the couple decided to terminate the pregnancy following detection of fetal abnormalities on prenatal sonography. The third fetus is also detected a distended bladder via ultrasonic screening in the first trimester and the pregnancy was terminated at 16 weeks of gestation.

### Targeted exome and Sanger sequencing results

We performed targeted exome sequencing on trio DNA samples from umbilical cord tissue of the second terminated fetus and the peripheral blood of his parents. An average sequencing depth of 158× was achieved, and 99.1% of targeted variants were covered at least to a 10× depth, and 98.9% of targeted variants were covered at least by 20×. The fetus-mother-father trio sequencing data were analyzed by Ingenuity Variant Analysis via entering the phenotype of megacystis. Compound heterozygous variants c.2051 G > A (p.R684H) (Fig. 1c) and c.3540_3541delinsTT(p.(E1180D, Q1181Ter)) (Fig. 1d) in *MYH11* (NM_001040114) in the index fetus were identified with high probability as causative variants with the recessive inheritance pattern. The mother was c.2051 G > A (p.R684H) heterozygous variant carrier, and the father was c.3540_3541delinsTT (p.(E1180D, Q1181Ter)) heterozygous variant carrier. Sanger sequencing validated the compound heterozygous variants. Amino acid 684 is in highly conserved protein region and is close to a complex salt bridge domain [20]. Amino acid 1180 and1181 are also highly conserved and Q1181Ter variant resulted in the termination of the protein. In silico variant prediction analysis predicted that the two variants most likely had damaging or diseasing-causing effects. According to standards and guidelines of the American College of Medical Genetics and Genomics and the Association for Molecular Pathology (ACMG/AMP) [21], c.2051 G > A(p.R684H) (PM2 + PM3 + PP3) was classified to be uncertain significance and c.3540_3541delinsTT(p.(E1180D,Q1181Ter)) (PVS1 + PM2) was classified to be likely pathogenic. Sanger sequencing targeting *MYH11* c.2051 G > A and c.3540_3541delinsTT was performed for the third fetus for the chorionic villus sample at 14 weeks, and the results were positive. Combining the prenatal ultrasonography results of megacystis with the Sanger sequencing results, the couple chose to terminate the third pregnancy.

### *MYH11* expression in the control and proband

Umbilical cord tissues from the control and proband were tested by Western blotting using anti-MYH11 antibody. Expression of the 250 kDa protein was detected in the samples. The proband MYH11 protein expression was markedly decreased compared with the control sample (Fig. 1e). These results support the hypothesis that compound heterozygous variants may disrupt the MYH11 protein expression and that its normal function may be damaged.

## Discussion

In the present study, targeted exome sequencing detected compound heterozygous variants, c.2051 G > A (p.R684H) and c.3540_3541delinsTT (p.(E1180D,Q1181Ter)), in *MYH11* (NM_001040114) in a Chinese family with MMIHS; the variants were maternally and paternally inherited, respectively. Western blotting study revealed that aberrant protein expression of *MYH11* may be caused by compound heterozygous variants, thereby reducing the contraction of smooth muscle to cause the clinical phenotype. Sanger sequencing confirmed that the second and third fetuses carried compound heterozygous variants, which were inherited from the parents, respectively. The first fetus was not available for molecular genetic testing, and it is supposed that the fetus inherited the compound heterozygous variants based on the abnormality of identical ultrasonic screening results with the subsequent two fetuses. The couple had no vascular smooth muscle diseases, and three fetuses with megacystis were all male. The previously reported female-to-male ratio of 2.4:1 may be due to a more severe form of the syndrome resulting in the shorter life span of male patients [4]. The termination of the pregnancy may be another factor contributing to the gender ratio. Currently, three reports have described the involvement of *MYH11* as AR inheritance in MMIHS family [11–13]. Our study reports compound heterozygous variants in *MYH11* from a nonconsanguineous family, which enriches the genetic spectrum and further emphasizes the AR inheritance of MMIHS.

*MYH11* encodes smooth muscle myosin heavy chain, which functions as a major contractile protein, and the variants have been reported to be associated with nonsyndromic thoracic aortic aneurysms and/or dissections (TAADs), patent ductus arteriosus (PDA), acute myeloid leukemia (AML), gastric and colorectal cancer, prostate and breast cancer, and bladder cancer [22–28]. Functional and genetic evidence has suggested that *MYH11* plays a role in carcinogenesis because it may function in cell migration and adhesion, intracellular transport, signal transduction, and cell proliferation [29, 30]. A knockout mouse model with disrupted smooth muscle myosin heavy chain showed MMIHS phenotypes, such as a thin-walled, giant bladder, and low intestinal mobility [31]. Actin and myosin are the two major contractile proteins that constitutes the smooth muscle. In addition to *MYH11*, *ACTG2, LMOD, MYLK*, and *MYL9*, relating to the smooth muscle contraction, have been reported to be involved in MMIHS. *ACTG2* mutations underlie a significant proportion of autosomal dominant MMIHS, and the other four genes are recently reported causative genes of AR MMIHS, which expands the genetic heterogeneity of the disease. A summary of the clinical and molecular findings of four AR genes is listed in Table 1. The genetic causes of MMIHS are heterogeneous and a more precise characterization of the phenotype and molecular investigation could contribute to the current understanding of this syndrome.

**Table 1:** Summary of clinical and molecular findings of four genes involved in autosomal recessive MMIHS

Gene	LMOD1	MYLK		MYL9		MYH11		
Patient number	1	2	1	1	1	1	1	2
Age	Neonate	Fetuses	Neonate	Neonate	Neonate	Neonate	7 years	Fetuses
Gender	F	1F/1M	F	F	M	M	F	M
Consangunity	+	+	+	+	+	-	-	-
Zygosity	Homo	Homo	Homo	Homo	Homo	Compound hetero	Hetero+CNV	Compound hetero
Variants	c.1108 C > T (p.Arg370*) (NM_012134.2)	c.3838_3844dupGAAAGCG (p.Glu1282_Glyfs*51) (NM_053025.3)	c.3985þ5C > A (NM_053025.3)	a deletion of 6964 bp (chr20:g.36548744_36555707del) (ENST00000279022.6)	c.3598 A > T (p.Lys1200Ter) (NM022844)	c.2809_2810del (p.Arg937Glyfs*7) (paternal) c.3422_3470del (p.Lys1141Thrfs*20) (maternal)	c.379 C > T (paternal) 1.3 Mb deletion in 16p13.11 (maternal) (NM_001040113.1)	c.2051 G > A (p.R684H) (maternal) c.3540_3541delinsTT(p.(E1180D,Q1181Ter)) (paternal) (NM_001040114)
Inheritance	Inherited from hetero parents	Inherited from hetero parents	Inherited from hetero parents	Inherited from hetero parents	Inherited from hetero parents	Inherited from hetero parents	Inherited from hetero parents	Inherited from hetero parents
Molecular testing	WES	WES	WES	WES	WES	WES	Sanger sequencing + arrayCGH	Targeted exome sequencing + Sanger sequencing
Ultrasonography	Prenatal + Postnatal	Prenatal	Postnatal	Prenatal+Postnatal	Prenatal	Prenatal+Postnatal	Prenatal+Postnatal	Prenatal
Megacystis	+	+	+	+	+	+	+	+
Hydronephrosis	+	−	+	+	−	−	−	+
Malrotation or obstruction of the intestine	+	−	+	−	−	−	−	−
Other phenotype	TPN, microcolon	Subcutaneous edema, oligohydramnios,respiratory distress	Polyhydramnios, microcolon	Microcolon, intestinal hypoperistalsis, mild mydriasis	Lung hypoplasia	Anhydramnios, dilated pupils, microcolon, ileal atresia, dilated esophagus	Microcolon, motor development delay, pupil dysfunction, growth hormone deficiency, central hypothyroidism	Oligohydramnios
Surgery	−	−	+	Not described	−	+	+	−
Outcome	Deceased	Pregnancy terminated/deceased	Deceased	Deceased	Deceased	Deceased	Alive	Pregnancy terminated
References	[8]	[9]		[10]	[11]	[12]	[13]	This report

+ present, − absent, *Hetero* heterozygous, *Homo* homozygous, *CNV* copy number variation, *TPN* total parenteral nutrition, *WES* whole exome sequencing

The prenatal diagnosis of MMIHS was challenging due to its limited ultrasonography findings. The commonest presenting feature of prenatal sonographic diagnosis is fetal megacystis or intra-abdominal mass which has been reported in more than 25 cases [4, 9, 11, 12]. The three male fetuses in our study were consistent with the previous studies that all observed with progressive distended bladder, and oligohydramnios was noted (AFI = 5.5 cm) in the second fetus. MMIHS can be confused with other prenatal structural anomalies with megacystis, such as posterior urethral valves (PUV), urethral atresia/stenosis, and prune belly syndrome (PBS). The prognosis of these diseases is different, and an accurate diagnosis between these anomalies could be useful for pregnancy decision-making or postnatal treatment. Targeted exome sequencing results in conjunction with the ultrasonic examination findings are sufficient for the diagnosis of MMIHS. The CVS Sanger sequencing results and the clinical phenotype in the third fetus further support the MMIHS diagnosis and confirm the AR inheritance in this family. A recent study for the prenatal differentiation of MMIHS from other posterior urethral valves by fetal urinalysis can be helpful in prenatal diagnosis of MMIHS according to a lower sodium and phosphoral level and a high calcium level [16]. Prenatal magnetic resonance during the second trimester can provide useful additional information in the small bowel, colon, and rectal contents, which assists in the diagnosis of MMIHS [32].

In conclusion, our study reports compound heterozygous variants segregating with MMIHS in a nonconsanguineous Chinese family, and these variants expands the mutation spectrum and reinforces the AR inheritance of this genetic heterogeneity syndrome. Insights into the genetic etiology of MMIHS are dependent on the causative gene study and further functional research. A defined single gene etiology facilitates genetic counseling relating to recurrence risk, opening avenues for preimplantation genetic diagnosis and future prenatal testing.
